# From Molecular Spacers to Conjugated Polymers in 2D Perovskites

**DOI:** 10.1021/acs.accounts.6c00210

**Published:** 2026-05-07

**Authors:** Brayan A. Martínez-González, Haydee Pacheco, Diego Solis-Ibarra

**Affiliations:** Laboratorio de Fisicoquímica y Reactividad de Superficies (LaFReS), Instituto de Investigaciones en Materiales, 7180 Universidad Nacional Autónoma de México, CU, Coyoacán , 04510 Ciudad de México, México

## Abstract

Two-dimensional (2D) organic–inorganic hybrid perovskites provide a stable alternative to three-dimensional (3D) absorbers, which often suffer from sensitivity to moisture and light. However, the traditional 2D perovskite architecture functions as a “quantum-well” structure, where insulating organic cations form dielectric barriers that restrict both light absorption and charge transport. The research described in this account focuses on transforming these passive organic spacers into active electronic components. Specifically, this transformation is achieved by incorporating diynes (molecules with two adjacent triple bonds) directly into the perovskite lattice and inducing topochemical polymerization through thermal treatment, which results in the formation of a 2D perovskite that intercalates a conductive polymer between its inorganic layers.

The incorporation of such a polymer brings drastic changes in the properties of these materials. For example, it can significantly reduce their bandgap by up to 1.5 eV, thereby moving absorption well into the near-IR (NIR) range. Similarly, it can also improve the conductivity of the resulting material by up to 3 orders of magnitude while also enhancing their hydrophobicity and overall stability.

In this Account, we describe the synthesis and characterization of these hybrid materials, highlighting how the inorganic lattice preorganizes diacetylene ligands to facilitate solid-state reactivity. Further, we discuss the impact of oxidative doping, showing that the incorporation of stable organic radicals in the polymers enhances electrical conductivity and the material’s absorption. We further establish the versatility of this strategy by expanding the library of diynes and halides, confirming that this approach is a robust and reproducible method for modifying the optoelectronic properties of various 2D perovskite scaffolds.

Beyond fundamental material design, we discuss the application of these systems in high-performance optoelectronic devices, specifically air-processed NIR photodetectors. For instance, devices utilizing one of these polymerized 2D-perovskites exhibit remarkable responsivities on par with state-of-the-art devices. Ultimately, this account argues that the integration of conjugated polymers represents a paradigm shift for 2D perovskites, successfully transforming the organic spacer from a passive dielectric barrier into an electronically active component, thereby opening the door to new and exciting properties and applications.

## Graphical abstract

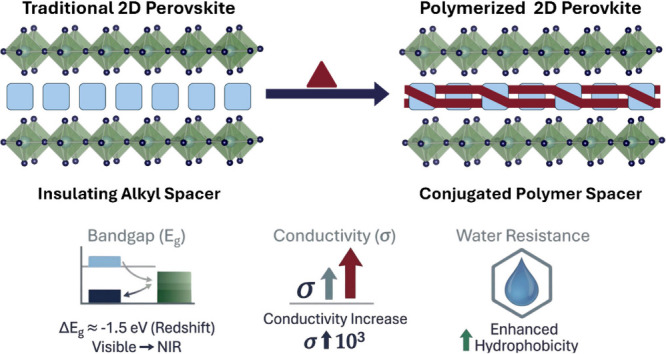

## Key References

-

Ortiz-Cervantes, C. ; Román-Román, P. I. ; Vazquez-Chavez, J. ; Hernández-Rodríguez, M. ; Solis-Ibarra, D. Thousand-fold Conductivity Increase in 2D Perovskites by Polydiacetylene Incorporation and Doping. Angew. Chem., Int. Ed. 2018, 57 (42), 13882 10.1002/anie.20180902830179301. The incorporation of conjugated diynes into 2D hybrid Pb perovskites is reported, with subsequent thermal treatment resulting in the formation of 2D perovskites that incorporate polydiacetylenes into their framework. Additionally, oxygen or iodine doping is shown to lead to the formation of stable radicals that increase conductivity by up to 3 orders of magnitude.^1^
-

Román-Román, P. I. ; Ortiz-Cervantes, C. ; Vasquez-Matias, J. I. ; Vazquez-Chavez, J. ; Hernández-Rodríguez, M. ; Solis-Ibarra, D. Incorporation of Conjugated Diynes in Perovskites and their Post-Synthetic Modification. ChemSusChem 2023, 16 (3), e202201505 36445827 10.1002/cssc.202201505PMC10108122. This report expanded the library of possible diynes and perovskite types. Six new perovskites composed of three distinct diynes are synthesized, all of which can be thermally polymerized to form conjugated polymers within the perovskite layers. Further, it is shown that the stability of the polymerized materials is enhanced and that the nature of the obtained polymer (and thus the material’s properties) differs from material to material.^2^
-

Zugasti-Fernández, D. ; Román-Román, P. I. ; Gutierrez-Avila, M. ; Gómora-Figueroa, A. P. ; Hernández-Cordero, J. ; Jancik, V. ; Hernández-Como, N. ; Solis-Ibarra, D. Air-processed, ultraresponsive NIR photodetectors using 2D perovskite hybrids. Chemical Science 2025, 16 (34), 15597 40756972 10.1039/d5sc02348cPMC12315253. Here is presented the new material: (PDA)_2_PbI_4_, which thermopolymerizes into (*poly*-PDA)­PbI_4_. The latter material is demonstrated to function as the active layer in high-performance NIR photodetectors, delivering responsivities up to 10^7^ A W^1–^ and external quantum efficiencies of 128% at 980 nm.^3^

## Introduction

The rapid growth of the global population and its dependence on technological advances have increased energy demand. Currently, fossil fuels continue to dominate and will remain the primary large-scale energy source in the foreseeable future.^4^ Energy is an essential requirement for the existence and development of human communities, and at a time when responsibility toward our planet and the environment is a priority objective, perovskites have emerged as promising light harvesters for photovoltaic energy.^5^

Three-dimensional (3D) perovskites have been recognized as promising light absorbers in solar cells; however, their sensitivity to moisture, which necessitates controlled processing conditions, represents a significant obstacle to successful commercialization.^6^ Two dimensional (2D) organic inorganic hybrid perovskites have recently attracted attention as viable alternatives to three-dimensional (3D) perovskite solar absorbers such as MAPbI_3_ (MA = methylammonium) and related materials.^7^ Compared with their 3D analogues, 2D perovskites demonstrated enhanced stability against water and light.^8^ This can be readily rationalized by considering the hydrophobic nature of the cations that replace volatile and hydrophilic methylammonium.^8,9^ However, the insulating nature of the organic cations in 2D perovskites also modifies the electronic structure of these materials, leading to reduced light absorption (larger bandgaps) and lower conductivities. This peculiar electronic structure comprising alternating semiconducting and insulating layers has been described as a quantum well structure.^10^ This electronic structure causes 2D perovskites to suffer from charge transport limitations and high exciton binding energy^11^ which in turn limits the power conversion efficiency of 2D perovskites compared to their 3D analogues.

Two-dimensional hybrid halide perovskites are formed by the alternation of inorganic perovskite layers and organic ligands. The way these two components interact determines the electronic structure of the material and, consequently, its optical behavior. In recent years, by increasing the conjugation of the organic ligands and reducing their electronic band gap, three main band-alignment schemes have been identified: type I [Figure 1, a)].^12,13^

**Figure 1:**
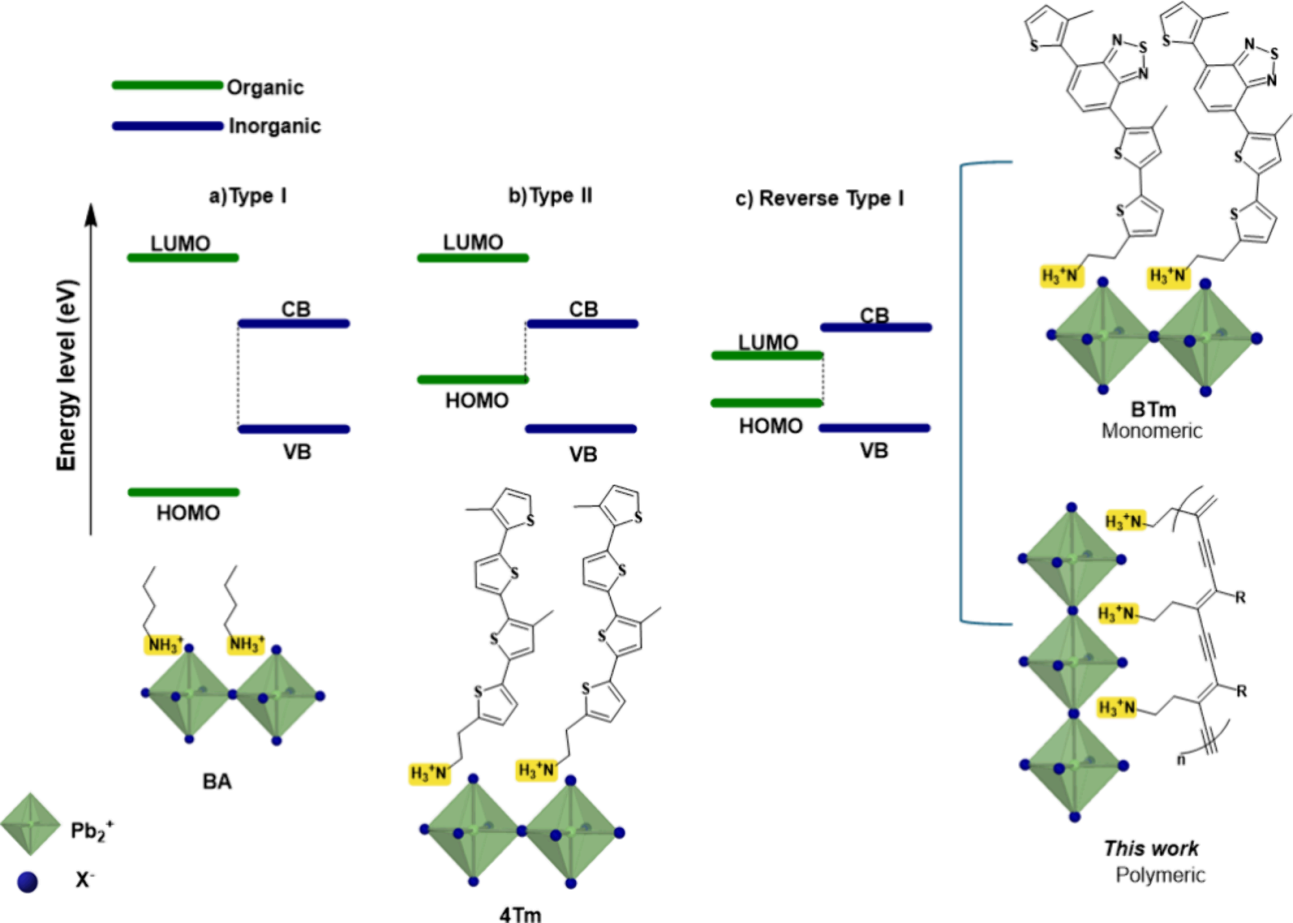
Excitonic structure and dynamics in different 2D hybrid halide perovskites. Three band-alignment schemes are illustrated: a) type I (CBM–VBM alignment), b) type II, and c) inverted type I (LUMO–HOMO alignment). In the classic Type I configuration (BA = n-butylammonium), the energy gap of the inorganic component is nested within that of the organic ligand; in this scenario, the organic frontier orbitals (HOMO and LUMO) act as potential barriers that confine charge carriers and excitons within the inorganic sublattice quantum wells. On the other hand, the Type II (4Tm = 2-(3‴,4′-dimethyl-[2,2′:5′,2″:5′′,2′′′-quaterthiophen]-5-yl)­ethylammonium) alignment exhibits a staggered band arrangement, where the energy offset between the phases promotes efficient spatial separation of electrons and holes, facilitating interfacial charge transfer. Finally, in the Reverse Type I regime (BTm = 2-(4′-methyl-5′-(7-(3-methylthiophen-2-yl)­benzo­[c]­[1,2,5]­thiadiazol-4-yl)-[2,2′-bithiophen]-5-yl)­ethylammonium), the energy hierarchy is inverted: the organic material’s bandgap is smaller and contained within the inorganic one, leading to carrier localization within the organic structure. This latter configuration, enhanced by the use of high dielectric constant ligands as discussed in this work, offers new pathways to modulate optoelectronic properties centered on the organic component’s functionality.

When examining the electronic response, it is not sufficient to focus solely on the difference between the excitonic gaps of the organic layers (HOMO–LUMO) and the inorganic layers; it is also important to consider the precise energetic alignment of these levels.^14,16^ Because both regions form separated layers, any mismatch between them can promote charge-transfer processes that ultimately influence the material’s absorption.^15^

While organic ligands have shown the ability to improve processability and enhanced stability against water and light, the vast majority of the devices and studies of 2D perovskites use a very limited of organic cations, and almost all of those have a low dielectric constant and thus, do not contribute to the optical or electronic properties of the materials. Since the early 2000s, pioneering efforts by Mitzi showed that the incorporation of oligothiophenes into 2D perovskites allow their behavior to be modified and their electronic structures to be gradually tuned.^17,18^ Later, Dou used a similar approach by introducing strongly electron-withdrawing groups together with a thiadiazole unit to modulate the electronic structure, which also enables the direct tuning of the optoelectronic properties. Recently, this group has also reported the incorporation of bidentate ligands into 2D perovskites, successfully solving the single-crystal structure of (MeX)­PbBr_4_ (MeX = 4,5-bis­(4-(2-ammonioethyl)­phenyl)-9,9-dimethyl-9H-xanthene) which confirmed that the bidentate ligands coordinate ipsilaterally to cation vacancies within the same inorganic layer, overcoming historical obstacles in lattice matching and ligand packing.^19–21^ Sargent demonstrated that the use of naphthalamides enables efficient charge transfer, although the structural disorder of these systems may limit their application in optoelectronic devices.^22^ In contrast, Stupp employed pyrene derivatives, showing that these cations contribute electronically to the band edges and influence charge carrier dynamics.^23^ All of these efforts, rely on highly conjugated monomeric species, however, those can be hard to synthesize, and are also constrained in size by the 2D perovskites scaffold. Thus, recently our group has devoted significant efforts to incorporate high-dielectric constant, conjugated polymers into 2D perovskites. [Figure 1, c].

In this account, we describe how the incorporation of conjugated diynes into 2D lead halide perovskites and the subsequent thermal treatment result in the topochemical formation of 2D lead-halide perovskites incorporating polydiacetylenes within their structure. The incorporation of polydiacetylenes in 2D perovskites, represents the first instance of a polymeric high-dielectric organic ligand in these materials. In turn, having polydiacetylenes in 2D perovskites induces significant changes in the properties of these materials, enhancing the stability, significantly enhancing the light absorption of these materials (particularly in the red and NIR region) and improving conductivity by up to 3 orders of magnitude. Further, we discuss the versatility of this approach and show that the use of diynes is a robust strategy applicable to various organic and inorganic scaffolds. Lat, we discuss the demonstrated and potential use of these materials in optoelectronic devices, particularly, in the fabrication of fully air-processable NIR photodetectors that retain stability for up to two months under ambient conditions.

The idea of incorporating polymers in perovskites, started with early studies by Tieke^24–26^ and co-workers, who demonstrated that irradiating 2D perovskites containing unsaturated organic cations can induce topochemical polymerization without disrupting the layered hybrid structure. However, their work focused mainly on cadmium chloride–based systems and diene-type monomers, which possess very wide bandgaps and give rise to polymers with limited electronic relevance.

Later, Takeoka and her group incorporated polydiacetylenes into lead halide perovskites, but the polymerization was triggered by γ radiation, which restricts practical applicability, further, no optical or electronic properties were studied at the time.^27^ Attempts have also been made to incorporate presynthesized conductive polymers into perovskites, although such methods often produce materials with low crystallinity and incomplete characterization.^28,29^ For this reason, our objective was to analyze how the incorporation of polydiacetylene and its subsequent doping affect the optical and electronic properties of 2D hybrid perovskites [Figure 2)].^1^

**Figure 2:**
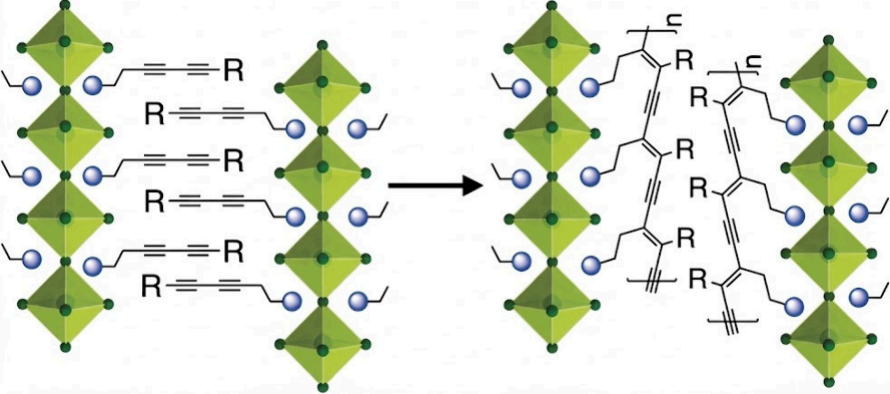
Representation of heat-induced polydiacetylene formation. Adapted with permission from ref 1. Copyright 2018 John Wiley and Sons.

Our initial hypothesis focused on the feasibility of introducing an *in situ* conductive polymer within the layers of a two-dimensional perovskite to substantially enhance its light-absorption and electrical-conductivity properties. For this purpose, the design of the organic molecule was crucial; we sought a ligand that was versatile, tunable, easy to characterize, accessible, low-cost, and fundamentally highly prone to solid state polymerization. These considerations led us to select the diacetylene family as the primary candidates. In the early stages of the research, synthesizing the diacetylene ligand represented a challenge due to issues related to precursor stability, scalability, and the multistep nature of the synthetic route required to construct these molecules. Later, we optimized the procedure to obtain it in significant quantities, overcoming these initial obstacles.

Once the ligand synthesis was resolved, we successfully incorporated it into a two-dimensional perovskite, specifically [DDA]_2_PbBr_4_, obtaining high-quality, optically transparent single crystals [Figure 3, c)]. Structural analysis of these crystals revealed a key finding: the diacetylene groups from adjacent ligands were stacked at very short intermolecular distances, around 3.8 Å [Figure 3, b)], a fundamental prerequisite for efficient solid-state topochemical polymerization.^30,31^ Our first attempt to induce polymerization was by irradiating the material with UV light; however, this method produced no observable change. In contrast, heating the material to 160 °C under an inert nitrogen atmosphere resulted in an immediate color change from transparent to reddish [Figure 3, d)]. Importantly, despite the color change, the crystallinity of the perovskite remained intact, while its absorption spectrum exhibited significant modifications [Figure 3, g)], suggesting the formation of a conjugated polymer.

**Figure 3:**
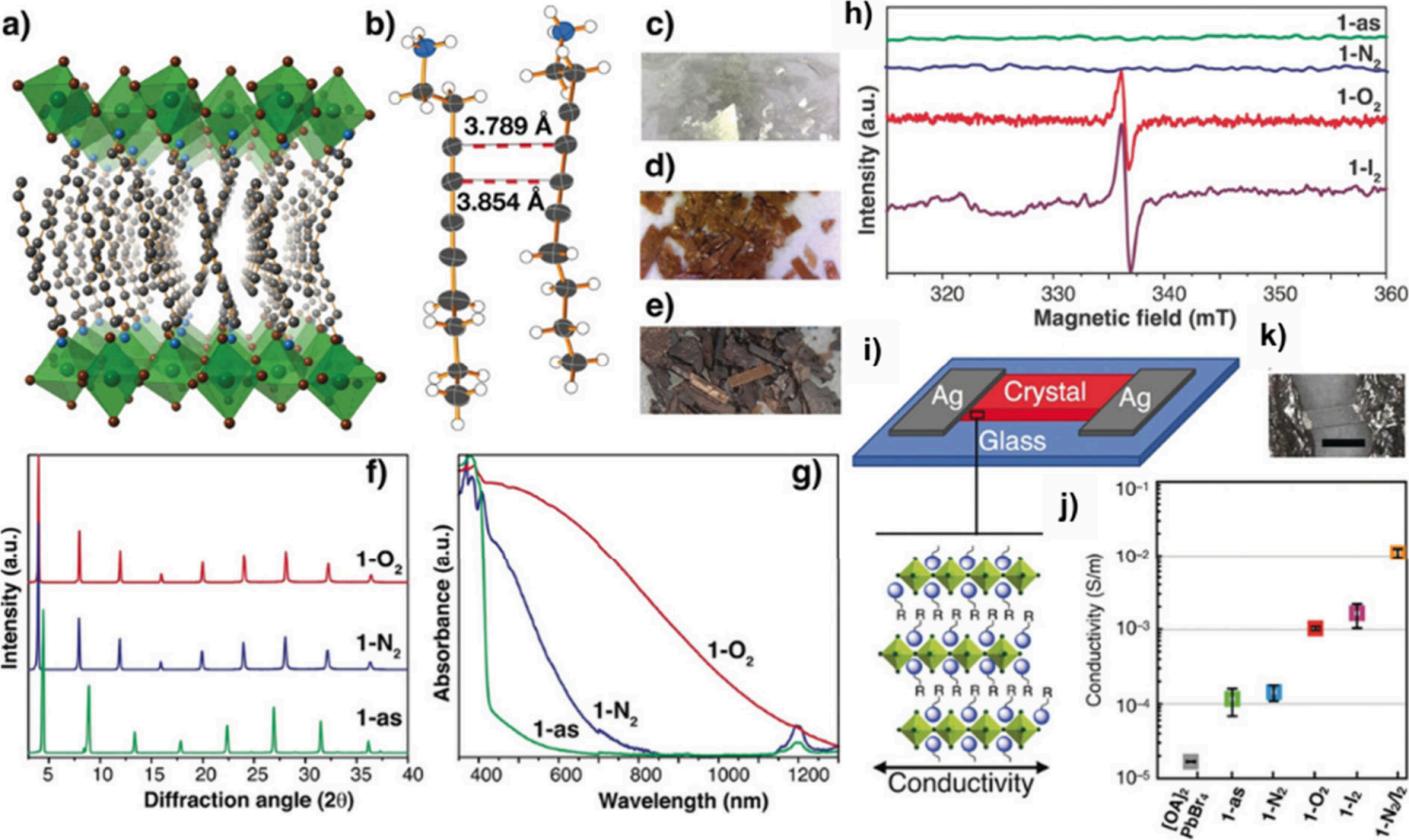
(a) Room-temperature single-crystal X-ray structure of as-synthesized [DDA]_2_PbBr_4_. Pb–Br octahedra: green, Pb: green, Br: brown, N: blue, C: gray. Hydrogen atoms are omitted for clarity. (b) Two neighboring DDA cations within [DDA]_2_PbBr_4_ crystal structure. Ellipsoids for non-hydrogen atoms are set at 50% probability. Dashed bonds highlight the two shortest intermolecular C­(sp) to C­(sp) distances. (c–e) Photographs of crystalline samples [DDA]_2_PbBr_4_ (c), [DDA]_2_PbBr_4_ in N_2_ (d), and [DDA]_2_PbBr_4_ in O_2_ (e). (f) Powder X-ray diffraction (PXRD) patterns. (g) UV/vis-NIR spectra of [DDA]_2_PbBr_4_, [DDA]_2_PbBr_4_ in N_2_, and [DDA]_2_PbBr_4_ in O_2._ (h) X-band electron paramagnetic resonance (EPR) of solid crystalline samples of [DDA]_2_PbBr_4_, [DDA]_2_PbBr_4_ in N_2_, [DDA]_2_PbBr_4_ in O_2_ and [DDA]_2_PbBr_4_ in I_2_ at 77 K. (i) The experimental setup used for single crystal electrical conductivity measurements and representation of the layered hybrid perovskites orientation showing the direction of conductivity measurements. k) Microphotograph showing a device made with a single crystal of [DDA]_2_PbBr_4_; scale bar: 4 mm. (j) Averaged values and standard uncertainties for in-plane conductivity of [OA]_2_PbBr_4_, [DDA]_2_PbBr_4_, [DDA]_2_PbBr_4_ in N_2_, [DDA]_2_PbBr_4_ in O_2_ and [DDA]_2_PbBr_4_ in I_2_, and [DDA]_2_PbBr_4_ in N_2_/I_2_ at 423 K. Adapted with permission from ref 1. Copyright 2018 John Wiley and Sons.

The polymerization temperature was determined based on thermal analysis (TGA/DSC) and optical absorption measurements. In the case of [DDA]_2_PbBr_4_, heating at 160 °C efficiently induces topochemical polymerization of the diacetylene units while remaining below the decomposition temperature of the material. Lower temperatures resulted in incomplete polymerization, whereas higher temperatures or prolonged heating led to partial degradation of the perovskite lattice. It is worth noting that said temperature changes depending on the specific material and at the time, it must be determined in a case-by-case manner.

The most significant and largely unexpected discovery occurred when we repeated this thermal treatment in air instead of under a controlled atmosphere. Under these conditions, the transparent crystals turned completely black [Figure 3, d)]. Surprisingly, crystallinity was once again preserved, but the change in optical properties was dramatic, with a pronounced increase in absorption extending into the near-infrared region. We hypothesized that, in addition to polymerization, the presence of oxygen was inducing oxidative polymerization, generating stable organic radicals within the organic layers of the structure. This represented a highly relevant and unanticipated finding. Encouraged by this discovery, we proceeded to test our hypothesis.^32^

Using electron paramagnetic resonance spectroscopy, we confirmed the presence of these organic-type radicals [Figure 3, h)]. We realized that having an *in situ* doped conductive polymer in the material would deeply influence macroscopic properties, so we designed an experiment to measure conductivity. To eliminate grain-boundary effects, we used single crystals of the polymerized material (in both N_2_ and air) and measured conductivity using silver (Ag) contacts [Figure 3, (i)]. The results were both satisfactory and revealing: the material treated in the presence of oxygen containing the free radicals showed an increase in conductivity of more than 1 order of magnitude compared to its counterpart polymerized in N_2_. Moreover, we demonstrated that the materials could be further and controllably doped through the introduction of I_2_, increasing conductivity by up to two additional orders of magnitude. This set of experiments provided fundamental evidence that effective doping does not occur in the inorganic lattice but rather resides in the organic component, directly influencing the enhancement of light absorption and electrical conductivity [Figure 3, j)]. In other words, when polymerization is carried out in the presence of O_2_, partial oxidation of the conjugated polymer occurs, leading to the formation of radical species along the polymer backbone. Evidence for this process includes the appearance of a broad absorption band extending into the near-infrared region (∼1200 nm), the detection of organic radicals by EPR spectroscopy (g ≈ 2.003, consistent with carbon-centered radicals in conjugated polymers), and a substantial increase in electrical conductivity. Mechanistically, oxidative doping generates polaronic states within the bandgap of the polymer, which enable low-energy electronic transitions responsible for the observed NIR absorption. Similar behavior has been widely reported in related conjugated polymer systems.^33,34^

In parallel, we observed that *in situ* polymerization of the diacetylene ligands had a tangible effect on both the surface hydrophobicity and the moisture stability of the materials, which, although related, are governed by distinct physical phenomena. Water contact angle reflects surface, whereas stability under ambient humidity is primarily controlled by water vapor permeability and by diffusion along grain boundaries and through the organic interlayer. To probe the former, contact angles were measured on films of [DDA]_2_PbCl_4_, [DDA]_2_PbBr_4_, [DDA]_2_PbI_4_, [NDA]_2_PbCl_4_, [NDA]_2_PbBr_4_, [PDTA]_2_PbCl_4_, and [PDTA]_2_PbBr_4_ deposited by spin-coating on PEDOT:PSS-coated glass substrates. An increase in hydrophobicity was observed for all heat treated samples, with the most pronounced change in [DDA]_2_PbI_4_ (from 68.2° to 85.8°). To assess moisture stability, [DDA]_2_PbCl_4_, [DDA]_2_PbBr_4_, and [DDA]_2_PbI_4_ were exposed to humid conditions (23 °C and 60% RH) and examined by X-ray diffraction. In all three cases the polymerized materials withstood these conditions markedly better than their nontreated counterparts an improvement we attribute to the reduced permeability of the polymerized organic layer, which hinders moisture ingress toward the inorganic framework. Thermal stability, evaluated by TGA and DSC under N_2_, was not significantly altered upon polymerization. Taken together, these results indicate that the polymerization process enhances both the surface hydrophobicity and, more importantly, the overall humidity stability of the materials.^2^

Despite the marked improvement in several properties, it is worth mentioning that up to date, we have not been able to obtain a single-crystal structure of the polymerized material. This can be understood by several facts. First, the stochastic nature of the polymerization results in a distribution of polymer sizes, and perhaps, slightly different chemical structures. Second, the polymerization yield is far from perfect, and thus, within the material, there is also, nonpolymerized diyne remaining; and third, on top of the significant distribution of organic fragments, those diffract significantly less than the heavy PbX_4_ layers, thus, significantly making it difficult to characterize by X-ray diffraction techniques. Despite the lack of an X-ray structure, there is substantial evidence that the postsynthetical treatment generates polymeric (or oligomeric) fragments, namely, the appearance of new double and triple C–C vibrations in the IR and ^13^C NMR spectrum and the identification of large polymeric fragments in digested samples of polymerized [DDA]_2_PbBr_4_ in the GPC.

To overcome the limitations in structural resolution arising from polymerization heterogeneity, an attractive solution to this problem could be the implementation of controlled polymerization via gradual thermal ramping, enabling the quantification of conversion through solid-state NMR or Raman spectroscopy. Furthermore, the use of Pair Distribution Function (PDF) analysis serves as a viable approach to characterize local disorder, facilitating the study of short-range order and providing a better understanding of the stochastic nature of topochemical polymerization within the crystalline lattice.

We also sought to determine whether this approach could be applied to other diacetylenes and to perovskites with different halides. To this end, we synthesized three cations and attempted to incorporate them into lead chloride, bromide, and iodide perovskites.^2^ This not only enables us to establish the actual scope of the phenomenon but also to better understand the polymerization process and its effect on the final properties of the materials.

Using two new ligands 2,4-nonadiyn-1-ammonium chloride (NDA) and 2,4-pentadiyn-5-trimethylsilyl-1-ammonium chloride (PDTA) together with the previously reported DDA, we successfully synthesized six lead-halide perovskites as polycrystalline powders: [DDA]_2_PbCl_4_, [DDA]_2_PbI_4_, [NDA]_2_PbCl_4_, [NDA]_2_PbBr_4_, [PDTA]_2_PbCl_4_, [PDTA]_2_PbBr_4_, [PDA]_2_PbI_4_ and (PDA)_2_PbBr_4_ [Table 1]. All were obtained in good yields by substituting bromide with chloride or iodide, and all were fully characterized, confirming in every case the formation of 2D halide perovskite structures. However, we were unable to isolate lead-iodide perovskites with NDA and PDTA, likely due to solubility issues associated with PbI_2_ under the conditions employed.^35^

**Table 1:**
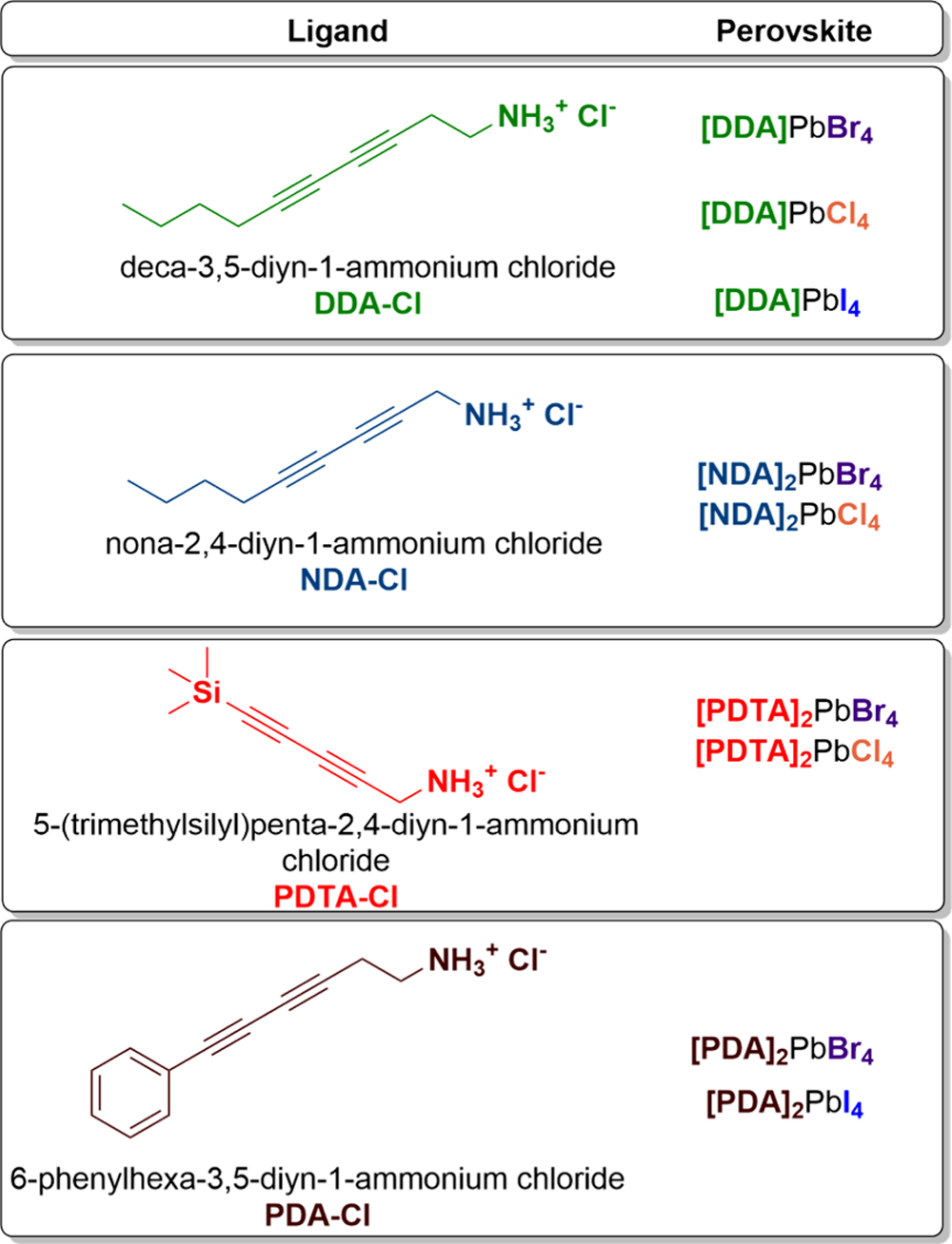
Summary of the Structures of Reported Dyines and Polymerizable 2D Perovskite Materials Derived from Them

We demonstrated that various diacetylene containing perovskites can undergo thermal polymerization to generate 2D perovskites incorporating polydiacetylenes within their inorganic layers. This confirms that the use of diacetylenes is a versatile and reproducible strategy applicable to a range of ligands and halides. By expanding the library of diacetylenes and precursor salts and employing more comprehensive characterization methods, we gained a deeper understanding of the polymerization process and its influence on the final properties of the materials.

In all cases, the materials underwent heat-induced polymerization, albeit to varying degrees, reflected in noticeable changes in their optoelectronic properties. Analysis of the resulting polymers confirms the formation of polyacetylenes and shows that their formation can vary widely in yield, chain length, polydispersity, and spin density. These variations collectively determine the final properties of each material. Although it is not yet possible to establish general rules to directly correlate the nature of the inorganic layer or the diacetylene structure to the properties of the resulting polymer, clear trends were observed. Most notably, chloride perovskites tend to form shorter polymers with lower spin concentrations compared with their bromide and iodide analogues.

Beyond the optoelectronic changes, we showed that the presence of polydiacetylenes can significantly increase the hydrophobicity and stability of 2D hybrid perovskites against moisture, as well as induce a marked increase in absorption extending into the near-infrared region.^36^

Motivated by the extended absorption of these materials, we explored this approach for the fabrication of NIR photodetectors, a range traditionally inaccessible to MHPs and MHP inspired materials. We synthesized 6-phenyl-3,5-hexadiynylamine and used it to prepare (PDA)_2_PbI_4_ by dissolving the free amine in ethanol and mixing it with a solution of PbI_2_ and HI. From this mixture, the material crystallizes as yellowish platelets. After a four-hour thermal treatment, the solid underwent a drastic color change, transforming into a black crystalline material.^3^ [Figure 4, a)].

**Figure 4:**
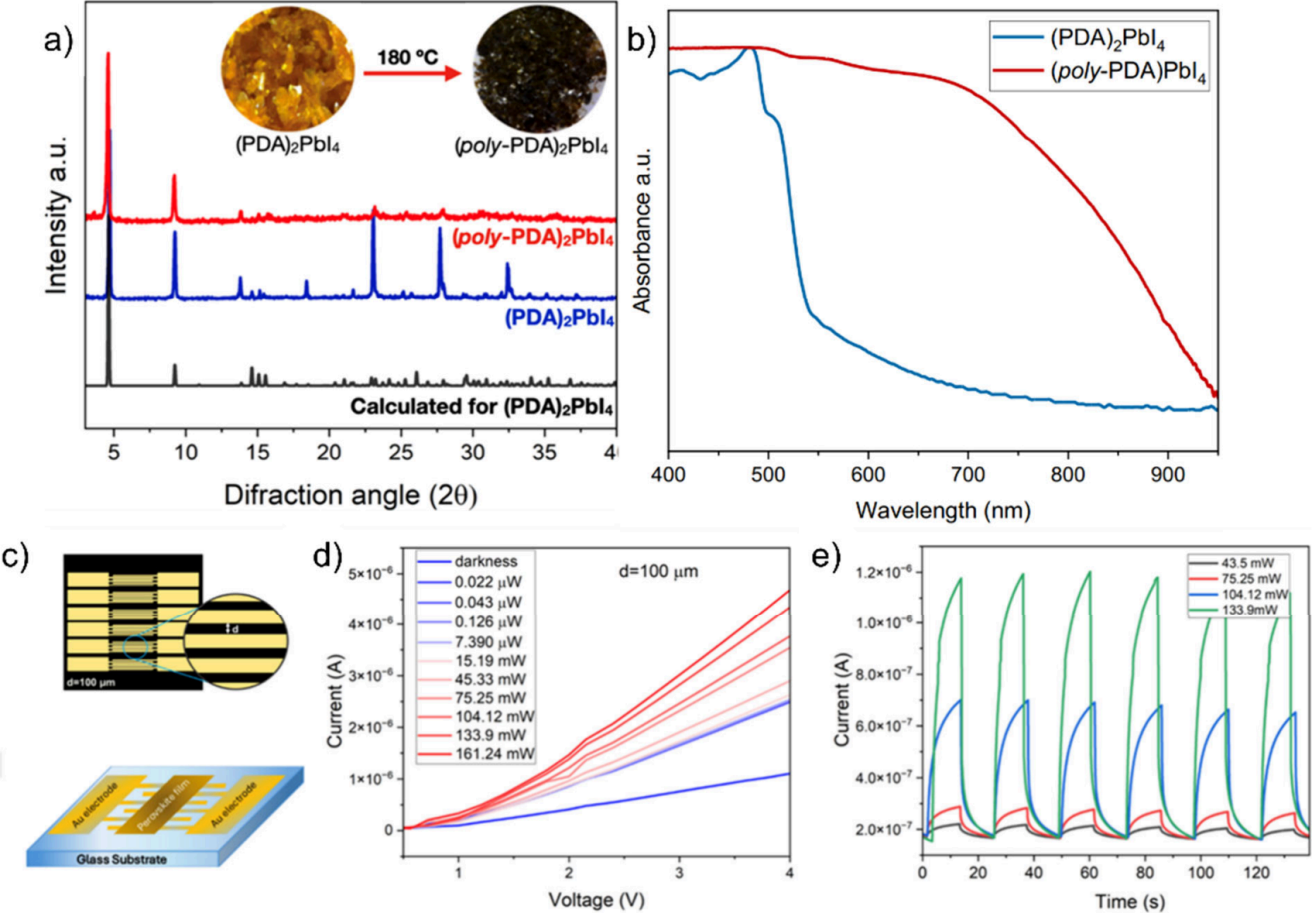
(a) Interdigitated electrode architecture and (c) and schematic device architecture. (b) UV–vis absorption, before and after heat treatment at 180 °C. (d) Current–voltage (*I*–*V*) characteristics of the photodetector at varying bias voltages and laser optical powers (at 980 nm) for an interelectrode spacing of 100 μm. (e) Temporal photoresponse of the photodetector under a 4 V bias voltage at different optical power levels (also at 980 nm). Adapted with permission from ref 3. Copyright 2025 Royal Society of Chemistry.

Single-crystal X-ray diffraction confirms that the diacetylenes remain intact in the precursor solid, with C≡C distances of 1.193 and 1.206 Å and C–C≡C–C angles near 177°. The structure also shows pronounced interdigitation of the PDA ligands and short C*sp*···C*sp* distances: 3.91, 3.86, and 3.85 Å, indicating a geometry that is favorable for polymerization.^30,31^

To study the effect of heating on optical properties, UV–Vis–NIR spectroscopy was performed before and after treatment. The initial material exhibits the typical absorptions of a 2D Pb–I perovskite, with a bandgap near 2.3 eV. After thermal treatment, the solid turns black, and its spectrum extends from the UV to the NIR, with a reduced bandgap of 1.2 eV. [Figure 4, b)].

Given this optical behavior, we evaluated polymerized (PDA)_2_PbI_4_ as the active layer in photodetectors. We fabricated devices with interdigitated gold electrodes (100 μm separation) on glass via photolithography [Figure 4, c)]. We optimized the process to enable polymerization of the material directly on the substrate, while preserving crystallinity and film integrity—critical factors for device performance. The films were deposited by drop-casting and gradually heated from 25 to 110 °C, a much lower temperature than that required for bulk samples (180 °C), as confirmed by UV–Vis–NIR spectroscopy.^37^

Device functionality was evaluated by irradiating with a 980 nm laser and measuring the dark current and the response under different light intensities^38^ [Figure 4, d)]. The best device based on polymerized (PDA)_2_PbI_4_ exhibited an average responsivity of 1.76 ± 0.048 × 10^7^ A W^1–^ (n = 4), comparable to advanced perovskite technologies such as MAPbBr_3_ single-crystal films,^39^ CsPbBr_3_–PbS^40^ heterostructures, or MAPb­(I_1–*x*_Br_*x*_)_3_ nanowire arrays, all operating at wavelengths below 780 nm.^41^ This value is also within the same order of magnitude as some of the best reported NIR photodetectors, such as Si/SiO_2_/graphene–PbS^42^ hybrid devices, In_0_._53_Ga_0_._47_As^43^ structures, or BODIPY-BF_2_-based phototransistors.^44^

The EQE at 980 nm reached 128.0 ± 3.6%, while the dark current measured at 4 V was 1.06 ± 0.019 × 10^–6^ A, which, although relatively high, is consistent with the device architecture.^45^ The temporal response [Figure 5, e)] showed a rise time of 6.73 s (10–90%) and a decay time of 2.74 s (90–10%), values that are reasonable given the polycrystalline nature of the films. Future work could incorporate more uniform deposition techniques, such as ultrasonic spray-coating, to improve crystal orientation and optoelectronic performance. Also, new device architectures and wavelengths shall be explored in the near future.

**Figure 5:**
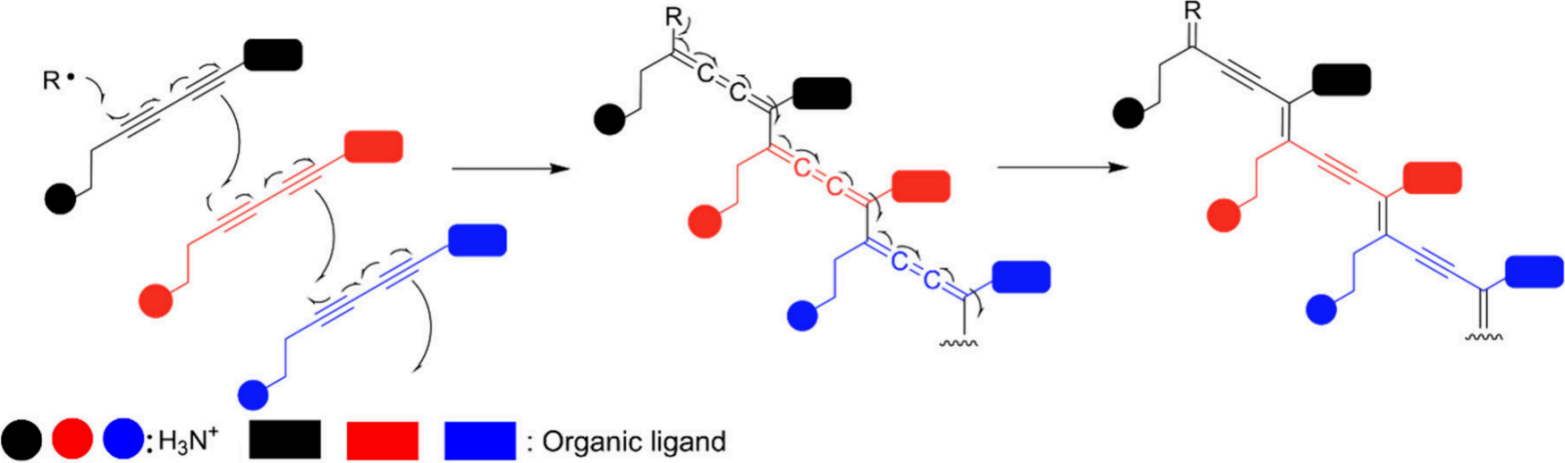
Radical-mediated topochemical polymerization of diynes in 2D perovskites. Schematic representation of the topochemical polymerization mechanism of diynes confined within the interlayer of a two-dimensional perovskite, where thermal or photoinduced activation promotes the formation of radical species on the C≡C bonds. These radicals, generated in a preorganized environment imposed by the inorganic lattice, couple in a controlled manner between neighboring diynes to yield conjugated polymer chains.

This set of observations naturally leads us to pose a more fundamental question: how does polymerization occur within the perovskite crystal lattice? At this stage, we do not aim to establish a definitive mechanism, but rather to propose an initial framework based on the accumulated experimental evidence. We start from the assumption that the perovskite not only confines the diyne monomers within the interlamellar space, but also spatially preorganizes them in a highly specific manner, thereby lowering kinetic barriers and channeling reactivity along well-defined crystallographic directions.^46^ From this perspective, polymerization can be understood as a collective response of the solid-state system, rather than as the sum of independent molecular events.^47^

However, the topochemical polymerization of diacetylenes occurs stochastically,^48^ which can result in polymers with different structures and, consequently, materials with varied properties and multiple potential applications. If this idea is correct, even small changes in structure should produce distinct products and appreciably modify their properties. A clear example is the identity of the halide in the lead perovskite, as it directly influences intermolecular interactions and distances, and therefore affects the behavior of the polymerized material.^49^

The reaction is initiated by thermal treatment, under which the monomeric units arrange into an ordered stack such that each monomer can react simultaneously with its two nearest neighbors. This geometric preorganization, imposed by the inorganic lattice, leads to a nearly parallel alignment of the diyne fragments and to short intermolecular distances between the reactive centers.^50^ In particular, the separation between C1_A_ and C2_B_ remains below 5 Å, reinforcing the notion that the topochemical polymerization of diacetylenes proceeds via an addition reaction favored by strict proximity and orientation criteria.^30,31^ As a consequence, the polymer chain that forms is oriented along a well-defined lattice direction.^51^ [Figure 5].

When diynes bear short and flexible aliphatic substituents, as in DDA and NDA, the molecules can accommodate more easily within the interlayer. This allows them to “fit” better into the available space, react more uniformly, and progress through polymerization without major steric hindrance, while maintaining good compatibility with the inorganic lattice. In contrast, aromatic substituents, as in PDA, render the molecule more rigid and electronically conjugated. This rigidity can be advantageous for stabilizing the polymer once formed, but it also introduces local strains that, if the crystal lattice cannot absorb them, may slow the reaction and limit the extent of polymerization.^52,53^ Finally, bulkier or strongly electron-donating substituents, as in PDTA, simultaneously affect the electronic distribution of the diyne and the way the molecules pack. In these cases, the reaction may become more controlled or even incomplete. Taken together, the substituents not only determine how reactive the diyne is, but also how effectively the perovskite can accommodate and sustain the structural changes associated with polymer formation.^54^

Under these conditions, thermal activation can induce the generation of organic radical species from the C≡C bonds of the diyne. These radical centers, stabilized by electronic delocalization along the conjugated system, can attack neighboring, prealigned monomers, promoting sequential radical coupling that leads to the formation of new C–C bonds.^55^ This propagation process progressively transforms the original triple bonds into a conjugated polymer chain of the polydiacetylene type, whose growth is guided and constrained by the crystal topology.

As mentioned previously, when the thermal treatment is carried out under an oxidizing atmosphere, such as O_2_, we not only observe a drastic color change in the material, but also a significant acceleration of the reaction. This behavior suggests that the presence of organic radicals favors both initiation and propagation, leading us to propose that polymerization involves radical intermediates and proceeds, at least in part, through a radical pathway. The reaction terminates when radical centers recombine or when local alignment is no longer favorable, resulting in the cessation of polymer growth.^56^

## Conclusions

The strategy of introducing preorganized diynes into 2D perovskites and promoting their topochemical polymerization constitutes a conceptually significant advance, as it transforms the organic spacer from a passive, dielectric element into an electronically active component. However, its technological potential depends on addressing a number of technical and fundamental challenges that remain unresolved.

The key to this transformation lies in the ability to induce *in situ* chemical reactions within the organic interlayer without collapsing the perovskite crystal lattice. In particular, the topochemical polymerization of diacetylene-based ligands demonstrates that the rigid preorganization imposed by the inorganic framework often regarded as a synthetic limitation can instead be exploited as an advantage to direct highly selective solid-state reactions. The fact that these reactions occur while preserving the structural integrity of the crystal suggests that 2D perovskites are not merely passive matrices but can serve as reactive environments capable of guiding chemical transformations with functional consequences. From this perspective, the organic spacer ceases to be a static component and is instead conceived as a dynamic chemical platform that can be activated thermally, photochemically, or chemically after crystallization.

The intrinsic variability of the topochemical process raises issues of reproducibility and heterogeneity: polymerization can generate distributions of chain lengths, varying degrees of local order, and domains with different spin densities, all of which influence both the density of states and carrier recombination pathways.

From a broader standpoint, these results suggest that the boundary between 2D perovskites and conjugated organic systems is more diffuse than traditionally assumed. By integrating conjugated polymer chains confined between inorganic layers, hybrid architectures are accessed that do not fit neatly into conventional categories of either organic or inorganic semiconductors. This intermediate character opens the door to emergent phenomena, such as unconventional band-alignment schemes, redistribution of excitonic confinement, and even alternative in-plane charge-transport pathways. In this context, the possibility of inverting or weakening the quantum-well character intrinsic to 2D perovskites represents a paradigm shift with direct implications for device design.

Regarding applications, the extension of absorption into the near-infrared and the substantial increase in *in-plane* conductivity make these materials exciting candidates for air-processable photodetectors and, potentially, for solar cells and as functional layers in 2*D*/3D photovoltaic architectures. Nevertheless, the transition from single crystals or proof-of-concept demonstrations to reproducible thin films and scalable devices remains a significant challenge. The compatibility of topochemical polymerization with other materials, such as methylammonium and organic transporting layers, will be decisive factors in assessing the true technological viability of this approach.
